# Transcriptional Regulation of Receptor-Mediated Mitophagy in Sunitinib-Resistant Renal Cancer Cells: Response to Succinic Acid

**DOI:** 10.3390/ph19091331

**Published:** 2026-08-24

**Authors:** Goksu Kasarci-Kavsara, Sinem Bireller, Baris Ertugrul, Bedia Cakmakoglu

**Affiliations:** 1 Department of Molecular Medicine, Aziz Sancar Institute of Experimental Medicine, Istanbul University, Istanbul 34093, Türkiye; 2 Department of Molecular Medicine, Institute of Graduate Studies in Health Sciences, Istanbul University, Istanbul 34126, Türkiye; 3 Department of Biochemistry, Faculty of Pharmacy, Acibadem Mehmet Ali Aydinlar University, Istanbul 34752, Türkiye

**Keywords:** sunitinib resistance, renal cell carcinoma, succinic acid, mitophagy, BNIP3/NIX/FUNDC1

## Abstract

**Background/Objectives:** Drug resistance is a major challenge in cancer therapy, and mitochondria contribute to this process by controlling both metabolic adaptability and cell survival signaling. Mitophagy, the selective lysosomal removal of dysfunctional mitochondria, has been implicated in therapy resistance, yet its role in sunitinib-resistant renal cancer remains poorly defined. **Methods:** In this study, acquired sunitinib resistance was established in ACHN renal cancer cells through eight months of stepwise dose escalation. Initial selection conditions were determined using CCK-8 viability and crystal violet colony assays in parental ACHN cells, whereas sustained proliferation under continuous sunitinib exposure was used as the operational criterion for the resistant phenotype. Resistant and parental sensitive cells were treated with 25 µM and 50 µM succinic acid, alone or in combination with sunitinib. Gene expression of BNIP3, NIX, FUNDC1, LC3, PINK1, Parkin, PGAM5, SRC, LONP1, and ATP5F1A was measured by RT-qPCR, and BNIP3 and NIX protein levels were assessed by ELISA. **Results:** Resistant cells showed significant upregulation of receptor-mediated mitophagy components BNIP3, NIX and FUNDC1 (*p* < 0.05), with no significant change in LC3, alongside suppression of PINK1, Parkin, and mitochondrial homeostasis-associated genes LONP1, PGAM5, and ATP5F1A (*p* < 0.05). Succinic acid predominantly reduced BNIP3 and NIX protein levels in both cell lines and suppressed BNIP3, NIX, and LC3 mRNA expression in resistant cells. In contrast, the sunitinib + 50 µM succinic acid combination selectively increased PARKIN, PGAM5, LONP1, and ATP5F1A expression in resistant cells (2.49- to 5.98-fold; *p* < 0.005), a pattern not observed in parental cells. **Conclusions:** These findings indicate that sunitinib resistance in ACHN cells is associated with upregulated transcription of receptor-mediated mitophagy components and downregulated transcription of PINK1/Parkin pathway genes, and that exogenous succinic acid selectively upregulates PARKIN and other mitochondrial homeostasis-related gene expression in resistant, but not parental, cells.

## 1. Introduction

Renal cell carcinoma (RCC) accounts for approximately 90% of kidney malignancies in adults, ranking fourteenth among all cancer types globally, with 434,840 new cases recorded in 2022 [1,2]. Age-standardized incidence rates are 7.2 per 100,000 in men and 3.6 per 100,000 in women, and the higher rates seen in high-income countries are generally attributed to better diagnostic and imaging infrastructure rather than a true biological gradient [1,3]. The most common histological subtype, clear cell RCC (ccRCC), which makes up 75–80% of all cases, is defined by inactivation of the von Hippel–Lindau (VHL) tumor suppressor gene and loss of chromosome 3p, with HIF-1α-driven angiogenic signaling representing the central pathophysiological feature of this subtype [4,5]. Around 25% of patients already have metastatic disease at the time of diagnosis, and up to 30% of those with initially localized tumors go on to develop distant metastases, leaving objective response rates in metastatic RCC at 15–25% and five-year survival below 5–10% [6].

Sunitinib malate is the standard first-line agent for metastatic RCC and works by competitively blocking the ATP-binding domains of multiple receptor tyrosine kinases (RTKs), including VEGFR-1/2/3, PDGFR-α/β, c-KIT, and RET [7,8]. Since its phase III trial in 2007, where it showed superior overall survival compared with interferon-α, sunitinib has remained the front-line treatment of choice for advanced disease by suppressing tumor angiogenesis [9]. The problem, however, is that most patients eventually develop acquired resistance, and this continues to be the main clinical barrier to long-term treatment success [10]. Resistance has been linked to several mechanisms—activation of alternative RTK pathways such as AXL, c-MET, and EphrinR2; lysosomal drug sequestration via ABC transporters; tumor microenvironment remodeling; and metabolic adaptation—though how exactly these mechanisms interact is still not fully understood [7,11,12].

Beyond its role in energy production through oxidative phosphorylation and the tricarboxylic acid (TCA) cycle, the mitochondrion is a multifunctional organelle involved in calcium signaling, reactive oxygen species (ROS) balance, intrinsic apoptosis, and lipid biosynthesis [13,14]. In cancer cells, mitochondrial reprogramming supports the kind of metabolic flexibility that includes the Warburg effect and helps cells adapt to the tumor microenvironment [15]. Sunitinib-related cytotoxicity has been reported to directly affect mitochondrial function by suppressing AMPK signaling, disrupting mitochondrial membrane potential, and triggering inner membrane permeability transition [16,17]. These observations implicate mitochondrial function in the development of chemotherapy resistance. Notably, sunitinib-resistant and sunitinib-sensitive cancer cells differ meaningfully in mitochondrial metabolism and quality control, consistent with a role for mitochondrial dynamics in drug resistance development [11,13,18].

Mitophagy, first described by Lemasters, is an evolutionarily conserved process by which damaged mitochondria are selectively delivered to lysosomes for degradation [19]. At the molecular level, mitophagy proceeds through two main pathways: the ubiquitin-dependent pathway, driven by the cooperative action of PINK1 (PTEN-induced putative kinase 1) and Parkin and triggered when inner membrane potential collapses following mitochondrial damage; and the receptor-mediated pathway, where LIR-domain-containing proteins on the outer mitochondrial membrane—BNIP3, NIX/BNIP3L, and FUNDC1—bind directly to LC3 without requiring prior ubiquitination [20,21,22]. These receptors are markedly upregulated under conditions such as hypoxia and energy deprivation, facilitating the uptake of damaged mitochondria into autophagosomes and their subsequent lysosomal clearance [23,24]. Beyond these two canonical pathways, two additional regulators deserve mention. The PGAM5 phosphatase modulates mitophagy flux through interactions with both PINK1 and FUNDC1, while the mitochondrial quality control protease LONP1 maintains TCA cycle integrity, oxidative phosphorylation, and mitochondrial protein homeostasis through its proteolytic activity [24,25].

Mitophagy plays a dual position in cancer biology, acting as a tumor suppressor in some settings and a tumor promoter in others [21,22]. In early-stage carcinogenesis, mitophagy is thought to limit genomic instability and contribute to apoptosis by clearing dysfunctional mitochondria and reducing mitochondrial ROS load. In advanced tumors, on the other hand, enhanced mitophagy may confer a survival advantage by reducing oxidative stress and allowing metabolic reprogramming, thereby supporting resistance to therapy [22]. Changes in BNIP3, NIX, FUNDC1, and LC3 expression have been shown to contribute to therapy-resistant phenotypes across several cancer models [26,27]. In renal cancer specifically, the GPD1L/PINK1/Parkin axis has been reported to suppress RCC progression, STAT2/SLC27A3/PINK1-mediated mitophagy has been implicated in pazopanib resistance, and mitophagy signaling has been more broadly associated with resistance to mTOR inhibitors [15,28,29]. These findings collectively highlight mitophagy as a potential therapeutic target in RCC, underscoring the need for strategies that specifically engage this pathway.

Succinic acid is a TCA cycle intermediate produced from succinyl-CoA and subsequently oxidized to fumarate by SDH, a reaction that contributes directly to the mitochondrial electron transport chain [30]. When SDH activity is impaired, intracellular succinate accumulates and stabilizes HIF-1α through PHD inhibition, creating a pseudo-hypoxic environment that suppresses epigenetic regulators including TET2—one of the central mechanisms linking TCA cycle dysfunction to oncogenesis [30,31,32]. Succinate also acts as an extracellular signaling molecule through SUCNR1 (GPR91), a G protein-coupled receptor whose activation initiates downstream responses including calcium mobilization, ERK1/2, and STAT3 signaling, thereby influencing tumor angiogenesis and inflammation [31,33]. The role of succinate in cancer biology is highly context dependent. Tumor-derived succinate has been shown in in vivo cancer models to promote macrophage polarization and epithelial-to-mesenchymal transition through the SUCNR1/PI3K/HIF-1α axis, facilitating cancer metastasis [34]. In colorectal cancer, *Fusobacterium nucleatum*-derived succinic acid was found in in vivo models to suppress the cGAS-IFN-β pathway, limiting CD8^+^ T cell trafficking into the tumor microenvironment and driving resistance to anti-PD-1 therapy; eradication of *F. nucleatum* with antibiotics reduced serum succinic acid levels and restored immunotherapy sensitivity [35]. Pharmacokinetic studies in mice have shown that following oral administration, succinic acid is rapidly distributed across tissues, with the highest concentrations recorded in the liver and substantial accumulation in the kidneys—a finding that may be particularly relevant given the high metabolic activity of renal proximal tubule cells [36]. Succinic acid derivatives have also been shown to exhibit selective cytotoxicity against multiple cancer cell lines at pharmacological concentrations and to suppress tumor growth and metastasis in zebrafish xenograft models in vivo [37]. Exogenously applied succinic acid appears to behave differently from endogenous oncometabolite accumulation, producing context- and concentration-dependent anticancer effects. Earlier work from our group showed that succinic acid induces in vitro apoptotic and antitumoral effects in endometrial cancer, T-cell acute lymphoblastic leukemia, and ACHN and CAKI-2 renal cancer cell lines, and that it can activate RIPK1-mediated necroptotic signaling in endometrial cancer [38,39,40,41]. Succinate released or applied in the extracellular space may influence mitochondrial morphology through specific membrane receptors and downstream signaling cascades [33]. Extracellular succinate has also been proposed to promote mitochondrial fission through its downstream AMPK axis [42], a process that under acute metabolic stress might redirect cells away from pro-survival mitophagy toward RIPK1-dependent death [41], though whether this operates in cancer cells remains to be established [42]. Given that PINK1-dependent mitophagy has already been directly implicated in TKI resistance in renal cancer, including pazopanib resistance mediated through STAT2/SLC27A3/PINK1 signaling [29], and that succinate accumulation is a direct upstream regulator of PINK1-dependent mitophagy through SDH activity, succinate metabolism represents a mechanistically plausible intervention point for TKI-resistant renal cancer rather than an arbitrary metabolic perturbation.

The existing literature on sunitinib resistance has not adequately addressed the relationship between mitophagy signaling and metabolic reprogramming. In particular, the role of changes in the balance between BNIP3/NIX/FUNDC1 receptor-mediated mitophagy and the PINK1/Parkin pathway in sunitinib resistance, and whether a TCA cycle metabolite such as succinic acid can modulate that balance, remains largely unexplored. To address this, we developed acquired sunitinib resistance in ACHN renal cancer cells through incremental drug exposure and examined how the gene expression profiles of receptor-mediated mitophagy components (BNIP3, NIX, FUNDC1, LC3), ubiquitin-dependent mitophagy regulators (PINK1, Parkin), and mitochondrial homeostasis-associated proteins (LONP1, PGAM5, ATP5F1A, SRC) change under resistance conditions, and how succinic acid treatment interacts with those profiles. The study characterizes sunitinib resistance in terms of a potential upregulation of BNIP3/NIX/FUNDC1-driven receptor-mediated mitophagy with concomitant suppression of PINK1/Parkin and LONP1/PGAM5/ATP5F1A. It further examines, for the first time using a comprehensive gene panel, whether succinic acid might modulate this adaptive response in a gene- and dose-dependent manner. We hypothesized that the mitochondrial adaptations underlying resistant renal carcinoma cells would be susceptible to exogenous metabolic intervention, motivating our investigation of succinic acid as a candidate modulator. We present this as an exploratory, hypothesis-generating study, designed to characterize transcriptional and steady-state protein patterns rather than to establish causal or functional mechanisms.

## 2. Results

### 2.1. Establishment of Sunitinib-Resistant Renal Cancer Cell Line

Sunitinib-resistant ACHN (rACHN) cells were generated through an eight-month stepwise dose-escalation protocol adapted from previously described methods. Prior to initiating resistance induction, the cytotoxic dose–response profile of sunitinib in parental ACHN (pACHN) cells was characterized by CCK-8 viability assay. The IC_50_ of sunitinib in pACHN cells was determined to be 26.27 µM. A crystal violet colony-formation assay was also performed to assess the effect of sunitinib on longer-term proliferative capacity. Residual colony formation was 63.4% and 53.71% at 12.5 µM and 25 µM sunitinib, respectively, relative to untreated controls. As 25 µM approximated the IC_50_ determined by the CCK-8 assay, prolonged exposure at this concentration caused a marked decline in cell viability during the induction period, rendering it impractical for sustained resistance selection. Accordingly, 10 µM sunitinib—a concentration that suppressed colony formation by approximately 40% while permitting residual cell survival—was selected as a sustainable concentration for continuing the dose-escalation protocol described below. Because resistance was induced under continuous chronic exposure rather than a single acute dosing interval, sunitinib concentrations were escalated stepwise from 1 to 20 µM -approaching, though remaining below, the acute 72 h IC_50_ established in pACHN cells -over a total induction period of 8 months. Before each increase in sunitinib concentration, cells were required to regain stable growth and maintain comparable morphology over three consecutive passages. The ability to maintain proliferation during chronic exposure to progressively increasing sunitinib concentrations was used as the operational criterion for the resistant phenotype. A formal post-selection IC_50_ comparison between pACHN and rACHN cells was not performed. Previous work in continuously exposed sunitinib-resistant renal cancer models indicates that changes in acute IC_50_ may not fully reflect the development of long-term proliferative tolerance [43]. Nevertheless, the absence of a matched post-selection dose–response analysis remains a limitation of the present study.

### 2.2. Sunitinib-Resistant ACHN Cells Exhibit Upregulated Expression of Receptor-Mediated Mi-tophagy Receptor Genes Alongside Downregulated Expression of PINK1/PARKIN Pathway Genes

**Figure 1:**
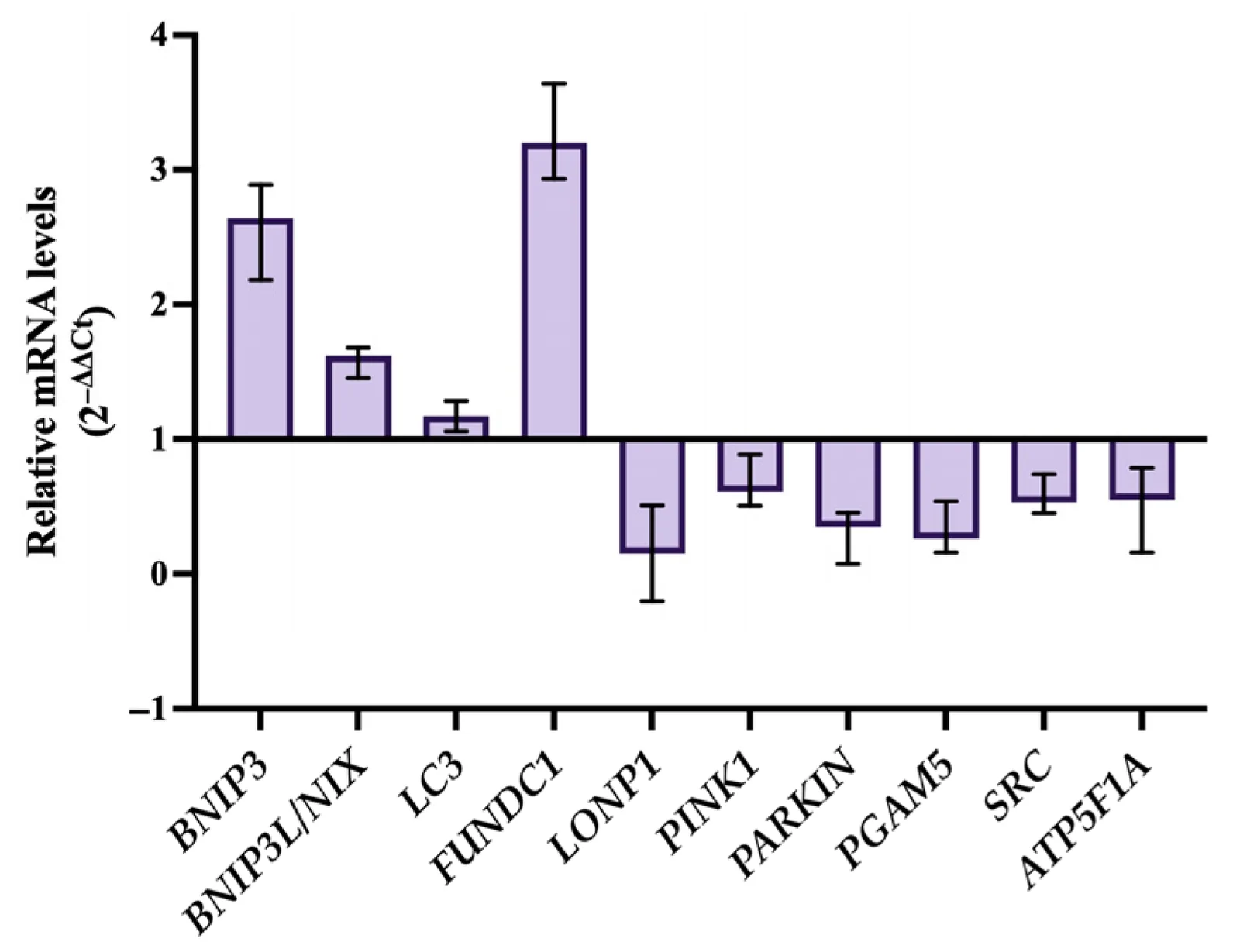
mRNA expression levels of mitophagy- and mitochondrial homeostasis-related genes in sunitinib-resistant (rACHN) cells relative to parental, sunitinib-sensitive (pACHN) cells. BNIP3, BNIP3L/NIX, and FUNDC1 were upregulated, whereas LC3 expression did not change significantly. PINK1/PARKIN pathway components and mitochondrial homeostasis-associated genes (LONP1, PGAM5, SRC, and ATP5F1A) were downregulated in resistant cells. The dashed line at 1 represents the expression level in parental cells (baseline, no change). Data are presented as mean ± SD (*n* = 3 replicates). Except for LC3, which showed minimal change, all other genes were significantly up- or downregulated in resistant relative to parental cells (*p* < 0.05).

To gain insight into the transcriptional reprogramming associated with acquired sunitinib resistance, we compared the mRNA expression profiles of mitophagy- and mitochondrial homeostasis-associated genes between untreated pACHN and rACHN cells (Figure 1). This analysis revealed a clear and functionally coherent divergence between the two cell lines, with receptor-mediated mitophagy genes and PINK1/PARKIN pathway components showing opposing directional changes. Receptor-mediated mitophagy genes were markedly upregulated in rACHN cells relative to pACHN controls. BNIP3, NIX, and FUNDC1 expression levels were significantly elevated in resistant cells (2.63-, 1.59-, and 3.17-fold, respectively; *p* < 0.05), while LC3 expression did not differ significantly between resistant and parental cells (1.10-fold; *p* > 0.05), suggesting that autophagosome membrane dynamics are not substantially altered at the transcriptional level during resistance acquisition. In contrast, genes encoding components of the PINK1/PARKIN-dependent ubiquitin-mediated mitophagy pathway were significantly downregulated in rACHN cells. PINK1 and PARKIN expression were reduced to 0.68- and 0.44-fold of pACHN levels, respectively (*p* < 0.05), consistent with reduced transcriptional support for kinase-dependent mitophagy signaling in the resistant state; whether this translates into impaired PINK1/Parkin pathway activity was not tested directly in this study. Genes associated with mitochondrial homeostasis and oxidative phosphorylation—LONP1, PGAM5, SRC, and ATP5F1A—were similarly suppressed in rACHN cells (0.21-, 0.44-, 0.58-, and 0.60-fold, respectively; *p* < 0.05).

### 2.3. BNIP3 and BNIP3L/NIX Protein Levels Differ Between Resistant and Parental ACHN Cells

**Figure 2:**
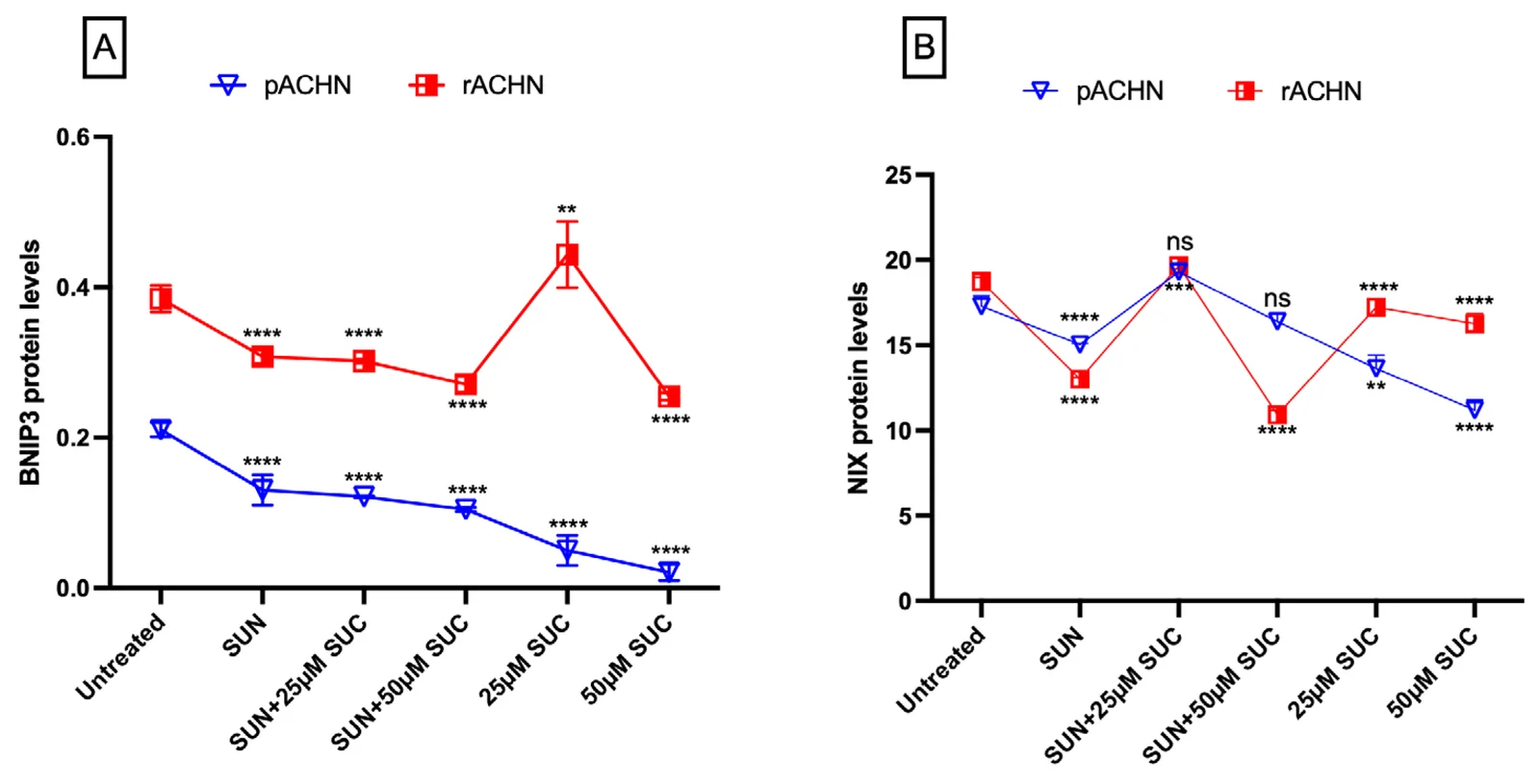
Effect of succinic acid (SUC) on BNIP3 (**A**) and NIX (**B**) protein levels in parental (pACHN) and sunitinib-resistant (rACHN) ACHN cells, measured by ELISA. Protein levels are shown following treatment with sunitinib (SUN) alone, SUN combined with 25 or 50 µM SUC, and SUC-alone (25 or 50 µM), relative to the respective untreated control within each cell line. Data are presented as mean ± SD (*n* = 3); lines connect group means to aid visual comparison between cell lines and do not indicate a continuous dose–response relationship. Asterisks denote statistically significant differences compared to the respective untreated control within the same cell line (** *p* < 0.01, *** *p* < 0.001, **** *p* < 0.0001); ns, not significant.

Comparison of protein levels between parental and sunitinib-resistant ACHN cells revealed a significant increase in BNIP3 protein levels in the resistant phenotype. Specifically, BNIP3 protein expression was significantly higher in rACHN cells than in pACHN cells (0.385 ± 0.018 vs. 0.211 ± 0.009 µg/mL; *p* = 0.016), indicating that the acquisition of sunitinib resistance is associated with constitutive upregulation of BNIP3 protein expression (Figure 2A). In contrast, BNIP3L/NIX protein levels did not differ significantly between the two cell lines (18.7 ± 0.2 vs. 17.3 ± 0.6 ng/mL; *p* > 0.05) (Figure 2B), suggesting that BNIP3L/NIX protein expression is not constitutively altered by the resistant phenotype. These findings indicate that BNIP3 and NIX are subject to distinct regulatory mechanisms in the context of sunitinib resistance, with BNIP3 exhibiting a resistance-associated baseline elevation that is not shared by NIX.

Succinic acid treatment suppressed BNIP3 protein levels in predominantly dose-dependent manner in both pACHN and rACHN cells, both as a single agent and in combination with sunitinib (Figure 2A), with one notable exception described below. In pACHN cells, the most pronounced reduction was observed with 50 µM succinic acid alone, which reduced BNIP3 protein to 0.021 ± 0.011 µg/mL—an approximately 10-fold decrease relative to the untreated control (*p* = 0.01) and a 6.2-fold decrease relative to the sunitinib-only group (*p* < 0.0001). In rACHN cells, succinic acid similarly suppressed BNIP3 protein in combination with sunitinib at both concentrations tested and as a single agent at 50 µM; however, single-agent succinic acid at 25 µM produced a significant increase in BNIP3 protein relative to untreated resistant control (0.443 ± 0.044 vs. 0.385 ± 0.018 µg/mL; Dunnett’s test, *p* = 0.0015), a nonmonotonic departure from the dose-dependent pattern observed elsewhere and not seen in pACHN cells. BNIP3 levels remained significantly higher in rACHN than in pACHN cells under matched treatment conditions (*p* < 0.05), with the exception of the 25 µM succinic acid combination group, indicating that resistance-associated elevation in BNIP3 protein is not fully overcome by succinic acid at the concentrations tested.

For BNIP3L/NIX, succinic acid exerted a suppressive effect on protein levels in both cell lines, with treatment-dependent divergence between rACHN and pACHN cells observed in several groups (Figure 2B). In rACHN cells, sunitinib alone significantly reduced NIX protein relative to the untreated resistant control (13.02 ± 0.1 ng/mL; *p* = 0.004 vs. pACHN), and the sunitinib + 50 µM succinic acid combination produced the most marked reduction (10.9 ± 0.2 ng/mL; *p* < 0.001 vs. pACHN). Single-agent succinic acid reduced NIX levels in pACHN cells in a dose-dependent fashion, whereas in rACHN cells this suppression was attenuated, particularly at 25 µM, where NIX levels remained comparable to the untreated resistant control (17.2 ± 0.4 ng/mL; *p* > 0.05 vs. control). The sunitinib + 25 µM succinic acid combination did not significantly alter NIX protein levels in either cell line relative to their respective controls (*p* > 0.05). Succinic acid therefore modulates BNIP3 and NIX protein expression in a dose- and context-dependent manner, with the resistant cell line exhibiting a blunted response, particularly for BNIP3—a pattern examined further below in relation to the corresponding transcriptional data (Section 2.4).

### 2.4. Succinic Acid Induces Distinct Transcriptional Responses in Mitophagy Related Genes Between Parental and Sunitinib Resistant ACHN Cells

**Figure 3:**
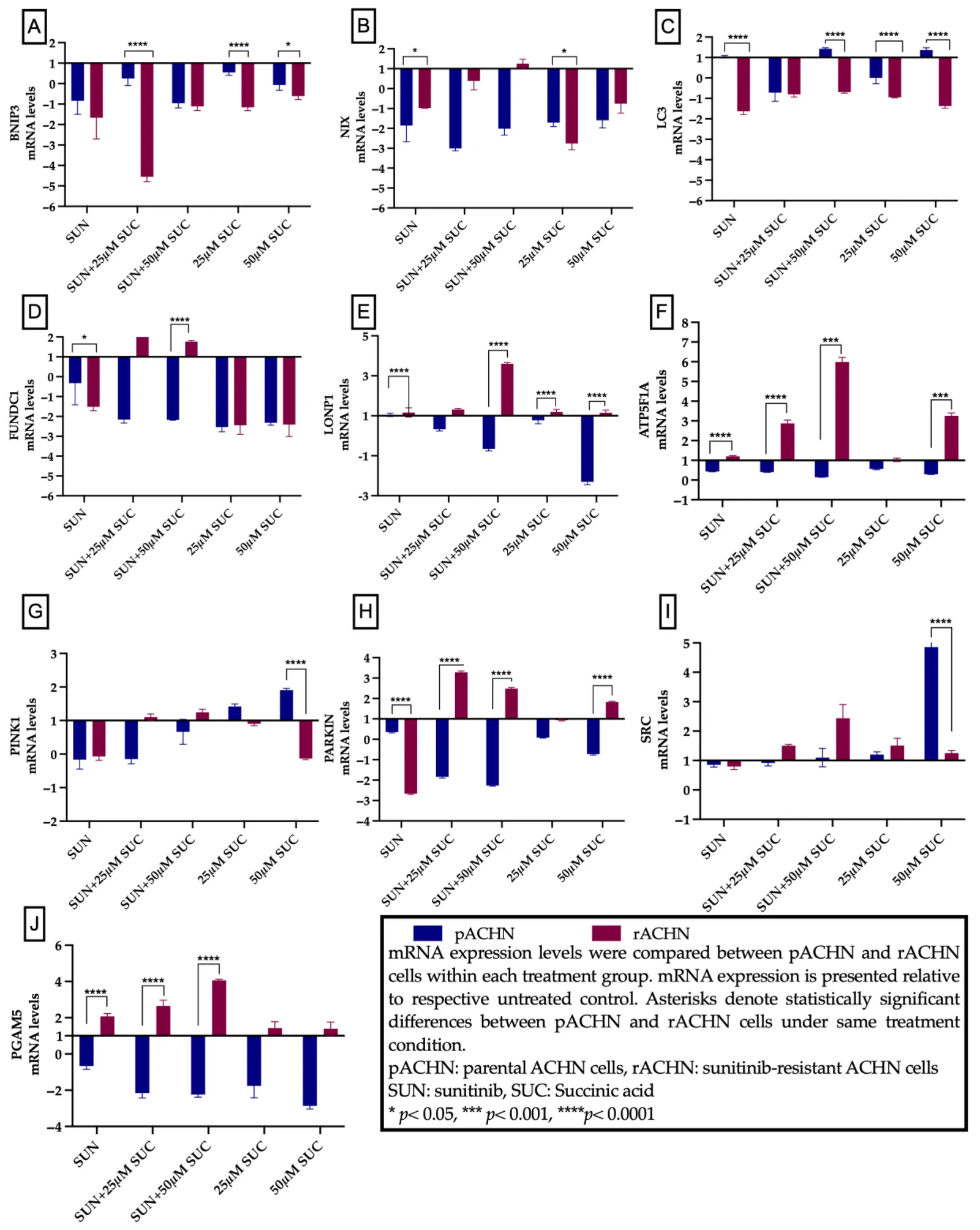
Relative mRNA expression levels of receptor-mediated mitophagy genes, PINK1/PARKIN pathway components, and mitochondrial homeostasis-associated genes in parental (pACHN) and sunitinib-resistant (rACHN) ACHN cells following treatment with sunitinib (SUN) alone, SUN combined with 25 or 50 µM succinic acid (SUC), or SUC alone (25 or 50 µM), relative to the respective untreated control within each cell line: (**A**) BNIP3, (**B**) NIX, (**C**) LC3, (**D**) FUNDC1, (**E**) LONP1, (**F**) ATP5F1A, (**G**) PINK1, (**H**) PARKIN, (**I**) SRC, (**J**) PGAM5. Data are presented as mean ± SD (*n* = 3). Asterisks denote statistically significant differences between pACHN and rACHN cells under the same treatment condition (* *p* < 0.05, *** *p* < 0.001, **** *p* < 0.0001).

In pACHN cells, sunitinib alone significantly suppressed PINK1, PGAM5, SRC, ATP5F1A, LC3, and PARKIN expression (*p* < 0.05), suggesting that sunitinib itself broadly perturbs the transcriptional regulation of mitophagy-associated genes independently of succinic acid (Figure 3). The addition of succinic acid further suppressed PARKIN, FUNDC1, PGAM5, and ATP5F1A in combination groups, while LC3 showed a mixed, dose-dependent pattern relative to sunitinib alone (further suppressed at 25 µM succinic acid, partially recovering at 50 µM). Notably, NIX was significantly suppressed across all succinic acid-containing groups in pACHN cells (Figure 3B)—including both combination and single-agent treatments—with the most pronounced reduction observed under sunitinib + 25 µM succinic acid (8.3-fold relative to sunitinib alone; *p* < 0.001). Single-agent succinic acid produced a concentration-dependent upregulation of PINK1 (Figure 3H) and SRC (Figure 3I), with 50 µM succinic acid inducing 3.74- and 4.86-fold increases, respectively (*p* < 0.05), while concurrently suppressing FUNDC1 (Figure 3D), PGAM5 (Figure 3J), and ATP5F1A (Figure 3F) at both doses. In sensitive cells, succinic acid therefore suppresses receptor-mediated mitophagy genes while partially restoring PINK1-dependent transcription, at least at the mRNA level.

In rACHN cells, the transcriptional response to succinic acid was qualitatively dis-tinct. BNIP3 expression was significantly suppressed in all succinic acid-containing treatment groups, both alone and in combination with sunitinib, though sunitinib alone did not significantly alter BNIP3 mRNA levels relative to untreated resistant controls (*p* = 0.213); this contrasts with the more variable pattern observed in pACHN cells (Figure 3A). LC3 was uniformly downregulated in rACHN cells under all conditions (Figure 3C), a pattern not observed in sensitive cells, suggesting a treatment-independent suppression of autophagosome-associated gene expression in the resistant context. The most striking divergence between cell lines was observed for PARKIN (Figure 3H), PGAM5 (Figure 3J), and ATP5F1A (Figure 3F) whereas these genes were suppressed by succinic acid in pACHN cells, they were markedly upregulated in combination treatment groups in rACHN cells—with sunitinib + 50 µM succinic acid inducing 2.49-, 4.06-, and 5.98-fold increases in PARKIN (Figure 3H), PGAM5 (Figure 3J), and ATP5F1A (Figure 3F) expression, respectively (*p* < 0.05). FUNDC1 showed a divergent, dose-dependent pattern in rACHN cells under combination treatment: expression was nominally increased relative to control at the lower succinic acid dose (Sun + 25 µM; not statistically significant) but significantly suppressed at the higher dose (Sun + 50 µM), in contrast to the consistent suppression observed in pACHN cells at both doses (Figure 3D).

To examine the relationship between transcript and protein responses, we compared BNIP3 and NIX mRNA and protein fold-changes across all treatment conditions in both cell lines (*n* = 20). A significant negative correlation was observed (Spearman’s ρ = −0.600, *p* = 0.0052), indicating that the mRNA and protein changes did not follow the same pattern across the conditions examined. This discordance may reflect post-transcriptional regulation, including differences in translation, protein stability, or degradation. While this analysis clarifies the relationship between transcript- and protein-level changes, it does not constitute a direct measure of mitophagy flux, which would require dedicated functional assays.

### 2.5. Exogenous Succinic Acid Selectively Upregulates PARKIN, LONP1, PGAM5, and ATP5F1A Expression in Sunitinib-Resistant Cells

In rACHN cells, sunitinib monotherapy markedly suppressed PARKIN expression (to −2.66 on the log2 scale shown in Figure 3H; *p* < 0.0001). Addition of succinic acid markedly increased PARKIN mRNA expression: the sunitinib + 25 µM succinic acid combination produced a 3.29-fold increase, and the sunitinib + 50 µM combination produced a 2.49-fold increase (*p* < 0.001 for both). Succinic acid applied alone at 50 µM also produced a 1.83-fold increase in resistant cells (*p* < 0.001). PGAM5 and LONP1 showed a comparable resistance-specific response. In rACHN cells, PGAM5 expression was elevated 2.07-fold under sunitinib monotherapy alone (*p* < 0.05), and succinic acid co-treatment amplified this further: 2.65-fold under the sunitinib + 25 µM combination and 4.06-fold under the sunitinib + 50 µM combination (Figure 3J, *p* < 0.005). LONP1 reached 3.6-fold under the sunitinib + 50 µM succinic acid combination (Figure 3E, *p* < 0.005). ATP5F1A followed the same pattern in rACHN cells (Figure 3F): the sunitinib + 50 µM succinic acid combination increased expression 5.98-fold (*p* = 0.001), and succinic acid alone at 50 µM produced a 3.25-fold increase (*p* = 0.007). In pACHN cells, ATP5F1A expression was reduced across all treatment groups. The concurrent upregulation of PARKIN, PGAM5, LONP1, and ATP5F1A in rACHN cells stood in contrast to the suppression of receptor-mediated mitophagy components—BNIP3, LC3, NIX, and FUNDC1—observed under the same treatment conditions.

## 3. Discussion

Mitophagy is the process by which aged, structurally compromised, or otherwise damaged mitochondria are selectively removed through a macroautophagic pathway, and it plays a key role in maintaining cellular homeostasis and organelle quality. Two general molecular routes drive this process. The ubiquitin-dependent pathway, mediated by the PINK1/Parkin axis, is initiated when inner mitochondrial membrane potential collapses under stress conditions; PINK1 accumulates on the outer mitochondrial membrane (OMM) and recruits the E3 ubiquitin ligase Parkin from the cytosol, activating it by phosphorylation to drive selective mitochondrial clearance [21,28]. The receptor-mediated pathway operates without ubiquitin or Parkin signaling; instead, BNIP3, NIX (BNIP3L), and FUNDC1—outer membrane proteins carrying LIR domains—bind directly to LC3 on the autophagosome membrane to initiate mitophagy engulfment [21,22].

**Figure 4:**
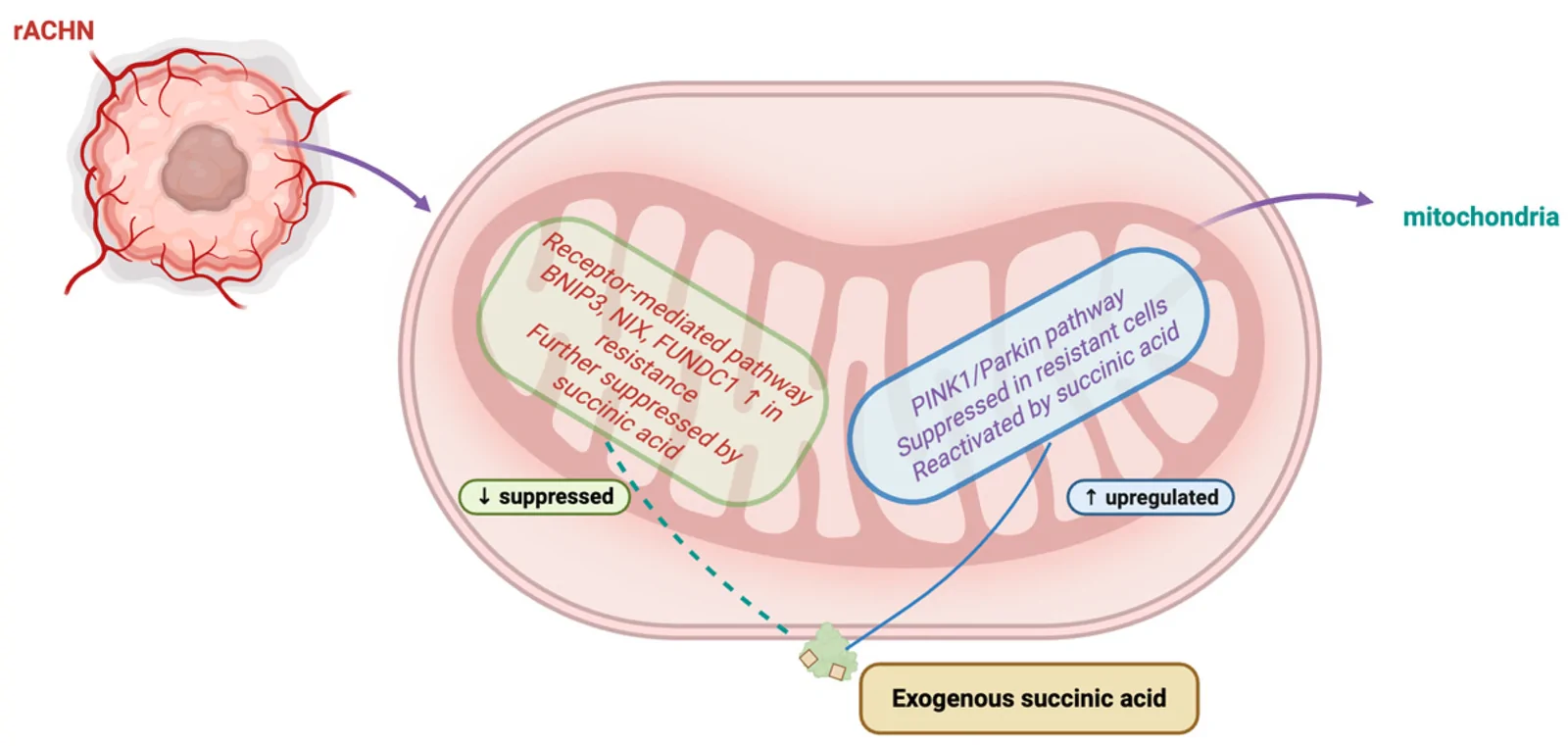
Schematic, hypothesis-generating representation—based on transcriptional data and not on direct measurement of mitophagy flux or pathway activity—of the opposing effects of exogenous succinic acid on gene expression within the two principal mitophagy pathways operating within mitochondria of sunitinib-resistant ACHN (rACHN) cells. Sunitinib-resistant ACHN cells (top left) exhibit mitochondria in which the receptor-mediated pathway, comprising BNIP3, NIX, and FUNDC1, is upregulated relative to sensitive cells, whereas the PINK1/PARKIN pathway is markedly suppressed. Exposure to exogenous succinic acid exerts opposing, gene- and dose-dependent effects on these two pathways. The receptor-mediated pathway (green capsule) undergoes further suppression following succinic acid treatment, as indicated by the dashed connector and the “↓ suppressed” label. In contrast, the PINK1/PARKIN pathway (blue capsule) is selectively upregulated at the transcript level by succinic acid, as indicated by the solid arrow and the “↑ upregulated” label. Arrow style and directional labeling together indicate the sign of the transcriptional response, independent of color rendering. (Created in BioRender. Bireller, S. (2026) https://BioRender.com/glbmbes (accessed on 3 August 2026)).

In this study, receptor-mediated mitophagy receptor genes were markedly upregulated at the transcriptional level in rACHN cells, while PINK1/PARKIN and mitochondrial homeostasis-associated genes were downregulated (Section 2.2; Figure 1); a schematic summary of this divergent regulation, and its modulation by exogenous succinic acid, is presented in Figure 4. SRC, a tyrosine kinase that can localize to the mitochondrial outer membrane and has been proposed to interact with components of receptor-mediated mitophagy, has also been linked to sunitinib resistance [7]; in our study, SRC gene expression responded to high-dose succinic acid in sensitive but not resistant cells, indicating that its role in this response differs by resistance status. Basal BNIP3 protein levels were higher in resistant cells, consistent with the mRNA findings (Section 2.3). It should be noted that elevated baseline expression of BNIP3, NIX, and FUNDC1 in rACHN cells does not, on its own, distinguish increased receptor-mediated mitophagy from other stress-associated transcriptional programs—including hypoxic signaling, oxidative stress, and broader metabolic adaptation—that are known to upregulate these same genes independently of mitophagy flux [21,22]; functional flux assays would be required to disambiguate these possibilities.

Mitophagy plays a dual role in cancer biology, depending on disease stage and the nature of cellular stress. In early carcinogenesis, clearing dysfunctional mitochondria and reducing mitochondrial ROS load may serve a protective function [21]. GPD1L has also been reported to suppress tumor spread in renal cancer models by activating the PINK1/Parkin axis [28]. Our data, however, show that cells under chronic drug pressure exhibit reduced PINK1/PARKIN gene expression alongside increased expression of BNIP3, NIX, and FUNDC1, a profile that may be associated with metabolic adaptation. This pattern is consistent with reports that receptor-mediated mitophagy becomes prominent during chemoresistance in solid tumors, with mitophagy receptor upregulation implicated in TKI stress adaptation [22,29]. FUNDC1 has also been shown to support chemotherapeutic resistance through the HIF-1α/BNIP3/FUNDC1 axis in endometrial cancer [24], indicating that this mechanism is not restricted to renal cancer.

When we looked more closely at LONP1 and PGAM5—two auxiliary regulators of mitochondrial quality control—both genes were found to be markedly suppressed in untreated rACHN cells compared with pACHN cells (*p* < 0.05). Mitochondrial Lon protease has been shown to accumulate at ER-mitochondria contact sites (EMC) under hypoxia, where it stabilizes the FUNDC1-ULK1 complex through its chaperone activity and initiates mitophagy [24]. At first glance, the suppression of LONP1 alongside an increase in FUNDC1 in the resistance condition appears paradoxical, but this pattern may reflect the distinct effects that acute hypoxic stress and chronic drug pressure exert on mitochondrial regulatory proteins. When succinic acid was applied in combination with sunitinib, this pattern changed markedly in resistant cells. LONP1 and PGAM5 expression increased in a dose-dependent manner in resistant cells upon succinic acid co-treatment (Section 2.5), consistent with a cumulative effect of drug pressure and metabolic intervention; the same combination suppressed both genes in sensitive cells, pointing to cell-type-specific effects of succinic acid on the LONP1/PGAM5 axis. PGAM5 interacts with both FUNDC1 and BCL-xL to influence the direction of mitophagy signaling, and while PGAM5 and FUNDC1 changed in a correlated, suppressed manner in sensitive cells, this correlation was lost in resistant cells, which may indicate that the regulatory relationship between these two genes differs under resistance conditions.

Regarding the interaction between mitophagy and succinic acid, intracellular succinate accumulation—as seen in chronic SDH deficiency—can upregulate BNIP3 and NIX transcription via PHD inhibition and pseudohypoxia, thereby promoting protective mitophagy [22,30,31]. In immune-metabolic contexts, the rate at which succinate is processed and the itaconate-mediated inhibition of SDH may also set the threshold for mitophagy activation in response to organelle damage signals [44]. Extracellular succinate has also been reported to promote mitochondrial fission through SUCNR1 and downstream AMPK signaling, which may serve as a prerequisite for mitophagy under certain conditions [33,42]. Beyond receptor-mediated signaling, extracellular succinate can also enter renal tubular cells directly via plasma-membrane dicarboxylate transporters such as SLC13A3, independently of SUCNR1 engagement [33]; SUCNR1 itself is expressed on renal proximal and distal tubular epithelial cells [33], making both a receptor-mediated and a transporter-mediated route plausible in ACHN cells. Distinguishing between these entry mechanisms—for example through SUCNR1 knockdown or transporter inhibition—was beyond the scope of the present study and is noted as a limitation. The transcriptional response of receptor-mediated mitophagy components to exogenous succinic acid was heterogeneous by gene and dose in resistant cells (Section 2.4), and the mechanistic basis of this pattern cannot be resolved from expression data alone; protein-level quantification or autophagic flux assays would be required. The consistent suppression of LC3 across treatment groups is noteworthy and might reflect an effect on autophagic flux, though the precise step affected could not be determined from expression data alone. PARKIN and ATP5F1A, by contrast, were markedly upregulated in resistant cells in response to succinic acid combinations (Section 2.5). Whether this reflects compensatory signaling in resistant cells or broader reorganization of mitochondrial quality control remains to be determined. Nonetheless, the overall pattern is compatible with exogenous succinic acid altering the mitophagy state in resistant cells in ways that affect cell death susceptibility. This is also consistent with our earlier observation in endometrial cancer, where succinic acid upregulated RIPK1 expression [41]. Whether mitophagy suppression and RIPK1 activation are functionally coupled in this setting remains an open question that warrants direct experimental testing. While succinic acid accumulates endogenously as an oncometabolite [31,32], exogenous application appears to produce different effects, though the precise role of TCA cycle reprogramming in this context requires further studies. Succinate derivatives have been reported to selectively affect cancer cells at pharmacological concentrations [45,46] and given the high mitochondrial density of renal proximal tubule cells, the interplay between succinate metabolism and mitophagy might be of relevance in ccRCC where SDH loss is common [31,32].

Overall, succinic acid’s effect on the mitophagy pathway in sunitinib-resistant ACHN cells differs from its effect in sensitive cells in a systematic way, and this difference may be related to mitochondrial adaptations that occurred during the development of acquired resistance. Succinic acid does not uniformly suppress mitophagy; rather, it produces a component- and dose-specific pattern of reorganization that diverges clearly between resistant and sensitive cells. These findings suggest that succinic acid might behave as a context-dependent metabolic trigger rather than a uniform mitophagy inhibitor, though this interpretation would require functional validation. A similarly divergent, cell type- and dose-dependent reorganization of this same mitophagy gene panel was recently reported by our group in malignant versus non-malignant cells exposed to a terpenoid-rich essential oil [47]. Heterogeneous, non-uniform engagement of receptor-mediated and ubiquitin-dependent mitophagy routes may therefore be a recurring feature of mitochondrial stress responses across bioactive-compound exposures, not a peculiarity specific to the sunitinib-resistant phenotype.

The observation that cytotoxic CD8+ T cells can secrete succinate autocrinally to enhance antitumor activity through SUCNR1 [48] indicates that the succinate–SUCNR1 axis may also be relevant to tumor–immune interactions, adding a further dimension to exogenous succinate signaling in the renal cancer microenvironment. However, the present 2D monoculture system inherently lacks immune and stromal components and cannot capture succinate-mediated paracrine signaling to CD8+ T cells or other tumor-infiltrating populations; this immunological dimension therefore remains untested here. These findings may be consistent with the dual role that mitophagy is known to play in cancer biology [28,29]. The complex, dose- and cell type-dependent gene expression pattern produced by succinic acid cannot be reduced to a single mechanistic explanation, and this complexity warrants further investigation in resistant cancer models. Xenograft or orthotopic renal tumor models with acquired sunitinib resistance represent a logical next step for assessing whether the mitophagy reorganization observed here translates into measurable changes in cell viability and drug response.

*Study Limitations:* This study examined mitophagy-related gene expression in sunitinib-resistant ACHN cells using a comprehensive panel and provided evidence that receptor-mediated mitophagy components are more prominently expressed in resistant cells, alongside suppression of PINK1/Parkin and mitochondrial quality control genes. These findings contribute to the understanding of the mitochondrial basis of sunitinib resistance independently of succinic acid. However, the findings are limited to a single cell line model, protein-level validation was restricted to BNIP3 and NIX, and the transcriptional and steady-state protein changes reported here do not distinguish increased mitophagy flux from impaired autophagosome–lysosome clearance; direct flux assays would be required to resolve this. Additionally, resistance was confirmed operationally through sustained proliferative capacity under continuous drug exposure rather than through a formal shift in acute IC_50_; methodological analyses in sunitinib-resistant renal cancer models indicate that IC_50_ is an unstable resistance indicator over prolonged continuous exposure, supporting sustained proliferation as a more consistent criterion [43], although direct comparison of acute dose–response curves between pACHN and rACHN would provide an additional, independent confirmation of the resistant phenotype. The markedly different gene expression response to succinic acid observed in resistant cells compared with sensitive cells indicates that this molecule engages distinct metabolic processes depending on resistance status, a question that in vivo models would be well placed to clarify. A future in vivo extension of this work could address these limitations, for example through a matched dose–response comparison between parental and resistant tumors, flux-based assessment of mitophagy alongside the transcriptional and steady-state protein measures reported here and expanded protein-level validation across the full gene panel. A further consideration is the translatability of the concentrations used here. Pharmacokinetic data in mice indicate that exogenous succinic acid is cleared rapidly (plasma t_1_/_2_ < 1 h) and has low oral bioavailability yet still reaches measurable concentrations in renal tissue shortly after administration [36]; whether the local exposure achieved with 25–50 µM succinic acid in vitro is sustained long enough in vivo to reproduce the transcriptional effects reported here remains to be established. This study also did not assess whether baseline expression of sunitinib’s primary receptor tyrosine kinase targets (VEGFR, PDGFR) was altered during resistance induction; established resistance mechanisms in RCC frequently involve activation of parallel or downstream signaling (e.g., alternative RTKs, angiopoietin/Tie2, tumor microenvironment remodeling) rather than changes in baseline receptor expression itself [10], but this was not directly examined in the rACHN model. The negative correlation observed between BNIP3/NIX transcript and protein fold-change further underscores that transcript-level data alone cannot be assumed to track functional mitophagic activity.

## 4. Materials and Methods

### 4.1. Chemicals and Reagents

Sunitinib malate (Mw:532.565 g/mol, Sigma-Aldrich, St. Louis, MO, USA) and succinic acid (Mw:118.09 g/mol, Sigma-Aldrich, USA) were used as the principal pharmacological agents in this study. Dimethyl sulfoxide (DMSO, Glentham Life Sciences, Corsham, UK), Dulbecco’s Modified Eagle Medium (DMEM, ATCC, Manassas, VA, USA), fetal bovine serum (FBS, ATCC, Manassas, VA, USA), and penicillin-streptomycin (Gibco Life Technologies, Grand Island, NY, USA) solution were used for cell culture procedures. Phosphate-buffered saline (PBS, Pan Biotech, Aidenbach, Germany), trypsin-EDTA solution (Sigma-Aldrich, USA), and L-glutamine (Sigma-Aldrich, USA) were used for routine cell maintenance. TRIzol reagent and bromoanisole (BAN) (Sigma-Aldrich, USA) were used for total RNA isolation, and isopropanol and absolute ethanol (Merck, Burlington, MA, USA) were used for RNA precipitation and washing. OneScript plus cDNA synthesis kit (Abm, Richmond, BC, Canada) and Luna Universal qPCR Master Mix (NewEngland Biolabs, Hitchin, UK) were used for reverse transcription and quantitative real-time PCR, respectively. The Cell Counting Kit-8 (CCK-8, Abbkine, Atlanta, GA, USA) was used for cell viability assays, and crystal violet powder (Glentham Life Sciences, UK) was used for clonogenic assays. Commercial enzyme-linked immunosorbent assay (ELISA) kits for BNIP3 (Cat. No. SEJ545HU, USCN, Wuhan, China) and BNIP3L/NIX (Cat. No. 201-12-5301, SunRed Biotechnology Company, Shanghai, China) were used for protein quantification. All chemicals, unless otherwise specified, were of analytical grade and obtained from commercial suppliers.

### 4.2. Cell Culture

The human renal cell carcinoma cell line ACHN, obtained commercially from the American Type Culture Collection (ATCC, USA), was used in this study. Cryopreserved stocks maintained in liquid nitrogen (−196 °C) from previous studies were thawed and expanded under standard cell culture conditions. Cells were cultured in DMEM supplemented with 10% FBS and 1% penicillin-streptomycin solution at 37 °C in a humidified atmosphere containing 5% CO_2_, without any drug treatment, until a sufficient cell number was obtained for subsequent experiments. Cell density and morphology were monitored daily by light microscopy. Culture medium was renewed every 2–3 days depending on cell density and medium color, and cells were subcultured by trypsinization upon reaching approximately 80% confluence.

### 4.3. Determination of Sunitinib Sensitivity by CCK-8 Assay

The sensitivity of parental ACHN (pACHN) cells to sunitinib was determined using the CCK-8 assay. pACHN cells were seeded into 96-well plates at a density of 1 × 10^5^ cells/well and allowed to attach for 24 h. Cells were then treated with increasing concentrations of sunitinib (0–100 µM) for 72 h at 37 °C, with five replicate wells per concentration. Following treatment, 10 µL of CCK-8 reagent was added to each well and incubated for 3 h. Absorbance was measured at 460 nm using a Multiskan microplate reader (Thermo Fisher Scientific, Waltham, MA, USA). Cell viability was calculated relative to untreated and DMSO-treated control cells.

### 4.4. Clonogenic Survival Assay

The long-term proliferative capacity of pACHN cells following sunitinib exposure was assessed using a crystal violet clonogenic assay. Cells were seeded into 6-well plates at a density of 1 × 10^3^ cells/mL (2 mL per well) and allowed to attach for 24 h. Cells were subsequently exposed to increasing concentrations of sunitinib for 72 h. Following treatment, wells were washed twice with PBS and stained with crystal violet solution (0.25 g crystal violet, 20 mL 95% ethanol, and 80 mL distilled water) for 20 min at room temperature in the dark. For quantitative analysis, stained colonies were solubilized in sodium citrate lysis buffer (0.1 M sodium citrate, 50% ethanol, pH 4.2 adjusted with HCl) for 30 min on an orbital shaker, and absorbance was measured at 570 nm.

### 4.5. Establishment of Sunitinib-Resistant ACHN Cells

Sunitinib-resistant ACHN (rACHN) cells were established through a chronic stepwise dose-escalation protocol consistent with previously described approaches for inducing acquired sunitinib resistance in renal cell carcinoma cell lines [11,43,49]. Briefly, resistance induction was initiated at the sublethal sunitinib concentration determined through preliminary cytotoxicity and clonogenic survival assays. pACHN cells were seeded in T25 flasks and initially exposed to 1 µM sunitinib for 72 h. Following drug exposure, the medium was removed and replaced with drug-free medium for a recovery period of 10–15 days, during which surviving cells were allowed to repopulate. This exposure-recovery design was intended to progressively select for stably resistant cells, as the subpopulation surviving drug washout is considered to comprise a mix of transiently tolerant and stably resistant cells; with repeated re-exposure, transiently tolerant cells are eliminated, and a stably resistant population is eventually enriched. Following each recovery period, cells were monitored daily, given medium changes every 2–3 days, and subcultured upon reaching approximately 70% confluence. Prior to each dose escalation step, cells were expanded for three passages and cryopreserved to maintain recoverable stocks at each resistance level. Cell growth was supported throughout with L-glutamine-supplemented medium, as drug-exposed cells exhibited substantially reduced proliferative capacity compared with untreated parental cells. Sunitinib concentration was escalated stepwise (1, 2, 5, 10, and 20 µM) over a total period of 8 months. At higher concentrations, where cell survival and proliferative capacity declined markedly, cultures were successively transferred from T25 flasks to 24-well and then 6-well plates to facilitate cell–cell contact within a smaller surface area and support repopulation; the untreated pACHN cells were maintained under the same plate-transition scheme to ensure comparable culture conditions. Cells that regained stable proliferative capacity at each concentration level were considered resistant to that dose, and the cycle was repeated at the next escalation step. The resulting rACHN cell line, maintained under continuous sunitinib treatment for more than 8 months, was used in all subsequent experiments.

### 4.6. Succinic Acid Treatment

Succinic acid was applied at 25 µM and 50 µM—concentrations previously shown to exert optimal cytotoxic activity in ACHN cells in our laboratory [40]—to both pACHN and rACHN cell lines. Cells were treated with succinic acid alone or in combination with sunitinib according to the experimental design. The experimental groups generated using these treatment combinations, which were used throughout the subsequent experiments, are summarized in Table 1.

### 4.7. BNIP3 and BNIP3L (NIX) Protein Level Assessment

Changes in BNIP3 and BNIP3L (NIX) protein levels were measured by sandwich ELISA in parental and sunitinib-resistant ACHN cells following treatment with sunitinib, succinic acid, or their combination. For BNIP3 quantification, cells were lysed in RIPA buffer and centrifuged at 13,000× *g* for 20 min at 4 °C; for BNIP3L (NIX) quantification, cell culture supernatants were collected and centrifuged at 3000 rpm for 20 min. All subsequent steps were performed according to the manufacturers’ instructions. Absorbance was measured at 450 nm using a microplate spectrophotometer, and protein concentrations were calculated from a standard curve and expressed as nanograms per milliliter.

### 4.8. Gene Expression Analysis

Following sunitinib and succinic acid treatment of pACHN and rACHN cell lines, expression changes in genes associated with mitochondrial quality control and mitophagy signaling pathways were assessed. Total RNA was first isolated using the TRIzol method and subsequently reverse-transcribed into cDNA using the OneScript Plus cDNA synthesis kit (Applied Biological Materials Inc., Vancouver, BC, Canada), according to the manufacturers’ instructions. The resulting cDNA samples were stored at −80 °C until quantitative real-time PCR (RT-qPCR) analysis. Target gene expression was assessed in synthesized cDNA samples using gene-specific primers [47]. GAPDH was used as the internal reference gene for normalization. Each experimental condition was represented by three independent replicates. Reaction mixtures were prepared according to the protocol in Table 2, and each biological replicate was additionally run in technical triplicate on 96-well plates. Plates were run on a Roche LightCycler 96 (Roche Diagnostics GmbH, Mannheim, Germany) Real-Time PCR instrument. Ct values obtained for target and reference genes were used to calculate relative gene expression as fold change.

### 4.9. Statistical Analysis

All statistical analyses were performed using GraphPad Prism version 11.0.2 (GraphPad Software, San Diego, CA, USA). Data are presented as mean ± standard deviation (SD) of three independent experiments. For each gene and protein target, differences between each treatment group and its respective untreated control within the same cell line were assessed by repeated-measures one-way ANOVA followed by Dunnett’s post-hoc test, with pACHN and rACHN analyzed as separate statistical families; the sole exception was BNIP3 mRNA expression in rACHN cells, for which Bonferroni-Sidak correction was used. Differences in treatment response between resistant and parental cell lines were assessed for each gene and protein using two-way ANOVA followed by Tukey’s post-hoc test across all pairwise comparisons. Family-wise error was controlled via each test’s built-in adjustment; a false discovery rate correction was not additionally applied. For BNIP3 and NIX, the only targets with paired transcript and protein data, the relationship between mRNA and protein fold-change across all treatment conditions and both cell lines (*n* = 20) was assessed using Spearman’s rank correlation. A *p*-value <0.05 was considered statistically significant.

## 5. Conclusions

Acquired sunitinib resistance in ACHN cells rests on a clear mitochondrial signature: the receptor-mediated mitophagy components BNIP3, NIX, and FUNDC1 rise, while the PINK1/Parkin axis and the quality-control regulators LONP1, PGAM5, and ATP5F1A fall. Exogenous succinic acid did not reverse this signature uniformly. Instead, it selectively restored PARKIN, LONP1, PGAM5, and ATP5F1A expression in resistant cells alone, leaving parental cells largely unaffected or suppressed at the same doses. This asymmetry is the central finding of the present work: succinic acid does not behave as a generic mitophagy modulator but as an agent whose activity is conditioned by the resistant phenotype itself. Mitochondrial quality control therefore emerges as a plausible, targetable axis in sunitinib-resistant renal cell carcinoma, and succinic acid as a candidate probe of that axis. Confirming this will require moving beyond transcript and steady-state protein measurements, through autophagic flux assays and, ultimately, in vivo models of acquired resistance. Consistent with the exploratory, hypothesis-generating framing of this study, these conclusions are intended to guide, rather than substitute for, the planned functional and in vivo validation.

## Figures and Tables

**Table 1:** Experimental treatment groups for pACHN (sunitinib-sensitive) and rACHN (sunitinib-resistant) cell lines.

pACHN (Sunitinib-Sensitive)	rACHN (Sunitinib-Resistant)
Non-treated (control)	Non-treated (control)
10 µM sunitinib (SUN)	10 µM sunitinib (SUN)
10 µM SUN + 25 µM succinic acid	10 µM SUN + 25 µM succinic acid
10 µM SUN + 50 µM succinic acid	10 µM SUN + 50 µM succinic acid
25 µM succinic acid	25 µM succinic acid
50 µM succinic acid	50 µM succinic acid

**Table 2:** Reaction components for RT-qPCR.

Reaction Component	Volume (µL)
Luna Universal qPCR Mix	10
Forward primer (10 µM)	0.5
Reverse primer (10 µM)	0.5
Template (cDNA)	<100 ng
Nuclease-free H_2_O	to 20

## Data Availability

The original contributions presented in this study are included in the article. Further inquiries can be directed to the corresponding author(s).
